# Garcinol in gastrointestinal cancer prevention: recent advances and future prospects

**DOI:** 10.1007/s00432-024-05880-6

**Published:** 2024-07-27

**Authors:** Nitika Patwa, Ritu Chauhan, Abhishek Chauhan, Manoj Kumar, Seema Ramniwas, Darin Mansor Mathkor, Adesh Kumar Saini, Hardeep Singh Tuli, Shafiul Haque, Petr Slama

**Affiliations:** 1 Department of Chemistry, Indian Institute of Technology, Delhi, India; 2 Department of Biotechnology, Graphic Era Deemed to be University, Dehradun, Uttarakhand, 248002 India; 3 Amity Institute of Environmental Toxicology Safety and Management, Amity University, Noida, U.P India; 4 Department of Chemistry, Maharishi Markandeshwar University, Sadopur-Ambala, 134007 Haryana India; 5 University Centre for Research and Development, University Institute of Pharmaceutical Sciences, Chandigarh University, Gharuan, Mohali, 140413 India; 6 Research and Scientific Studies Unit, College of Nursing and Allied Health Sciences, Jazan University, Jazan, 45142 Saudi Arabia; 7 Department of Bio-Sciences and Technology, Maharishi Markandeshwar Engineering College, Maharishi Markandeshwar (Deemed to Be University), 133207 Mullana, Ambala, India; 8 Gilbert and Rose-Marie Chagoury School of Medicine, Lebanese American University, Beirut, 11022801 Lebanon; 9 Laboratory of Animal Immunology and Biotechnology, Department of Animal Morphology, Physiology and Genetics, Faculty of AgriSciences, Mendel University in Brno, 61300 Brno, Czech Republic

**Keywords:** Garcinol, Gastrointestinal cancers, Pharmacokinetics, Apoptosis, Targeted therapy

## Abstract

Gastrointestinal cancers continue to pose a significant global health challenge, with millions of new cases diagnosed each year. Despite advancements in treatment, the prognosis for many patients remains poor. This article explores the potential of garcinol, a polyisoprenylated benzophenone found in various *Garcinia* species, as a therapeutic agent against gastrointestinal malignancies. The objective is to review recent research on garcinol’s anticancer properties, its mechanisms of action, and safety aspects. Garcinol exhibits anticancer effects in esophageal, gastric, colorectal, pancreatic, and liver cancers by inhibiting metastasis, inducing apoptosis, and targeting key molecular pathways in cancer progression. Nanotechnology is explored as a means to enhance garcinol delivery and efficacy. Safety assessments suggest a promising toxicity profile. Garcinol shows significant potential as a natural therapeutic agent for gastrointestinal cancers, and future research is needed on optimizing its delivery, exploring synergistic combinations, and conducting clinical trials to validate its efficacy and safety for clinical applications.

## Introduction

Cancers are characterized by aberrant cell proliferation that can infiltrate or spread to other bodily regions. Cancer continues to rank among the world’s most terrible diseases, even with ongoing improvements in diagnostic and treatment techniques (Cao et al. 2021). Currently, which therapeutic methods are used depends on the type and stage of the malignancy. Among the various treatment options include chemotherapy, radiation therapy, targeted therapy, and surgery (Kaur et al. 2023). According to projections, there will be approximately 26 million additional cases of cancer by 2030. As per 2018 report, there were 3.4 million associated fatalities and an anticipated 4.8 million new cases of gastrointestinal (GI) malignancies worldwide (Wang et al. 2024). In the world, 1 in 4 cancer diagnoses and 1 in 3 cancer-related fatalities are caused by gastrointestinal (GI) cancers. Numerous genetic and environmental factors may influence the development and risk of gastrointestinal malignancies, according to studies (Lu et al. 2021). Natural remedies have several special advantages in modern medicine as compared to conventional chemotherapeutic medications. These advantages include low cost, low toxicity, high patient acceptance rate and minimal side effects.

Natural phytochemicals are a rich source of innovative medications for a wide range of illnesses. It has been demonstrated that these substances, which are thought to be safe for human usage, alter important cellular signaling pathways that cause them to have anticancer properties (Sheikh et al., 2020). *Garcinia indica/ Garcinia cambogia* fruit rinds naturally contain a polyisoprenylated benzophenone called garcinol. Numerous investigations using cancer cell lines and experimental animal models have demonstrated the anti-cancer effects of garcinol (Aggarwal et al. 2020; Noreen et al. 2023). Furthermore, garcinol has been demonstrated to have powerful anticancer effects, activating apoptosis and autophagy, and lowering tumor cell resistance to chemotherapeutic agents in gastric cancer. Old conventional and newly developed anticancer agents face challenges due to the development of drug resistance in cancer cells, which is intricately linked to epigenetic alterations. Epigenetic changes (such as DNA methylation and histone acetylation), decrease the expression of target enzymes while increasing the expression of drug export pumps, resulting in drug resistance (Asano 2020).

Cancer immune evasion is yet another significant challenge in cancer treatment. Tumors cells use mechanisms to block immune checkpoints, such as PD-1, PD-L1, and CTLA-4, and further block proliferation and activation of T-cell. They also attract immunosuppressive cells to form a protective environment around themselves. Additionally, within the tumor microenvironment (TME), tumors accumulate specific metabolites and signaling factors that inhibit function of immune system. They also reduce the availability of vital nutrients to immune cells, further weakening the immune response (Dutta et al. 2023; Wang et al. 2023; Kim and Cho 2022; Kallingal et al. 2023). Metabolic reprogramming, including the Warburg effect, enables cancer cells to survive under various conditions. Cancer cells frequently develop resistance to medicines by processes such as drug efflux and changes in target genes, complicating treatment even more (Liberti and Locasale 2016).

This review delves into the intricate mechanistic insights of the anticancer aspects of garcinol, providing an up-to-date exploration of recent trends and advancements in its research and application. This comprehensive review aims to offer a holistic comprehension of garcinol’s therapeutic potential and its diverse implications for drug discovery and development.

## Methodology

We conducted comprehensive searches using PubMed, Scopus, and Google Scholar to gather relevant studies. Relevant full-length articles published in peer-reviewed journals from January 2005 to April 2024 were included. Followings were inclusion and exclusion criteria’s.

### Inclusion criteria

- Studies involving animal models or in vitro experiments on specific type of cancer (Esophageal/Gastric/Colorectal/Pancreatic / Liver/ Gallbladder).
- Research examining the effects of garcinol, either alone or in combination with other molecules.
- Preclinical studies (in vitro and in vivo) reporting on biological outcomes (e.g., tumor growth inhibition, apoptosis induction), or molecular outcomes (e.g., changes in gene/protein expression).
- Studies published in English.

### Exclusion criteria

- Studies not specifically focused on our cancer of interest (e.g., general studies on other types of cancer or mixed populations without separate analysis for specific cancer type).
- Studies not investigating the effects of garcinol.
- Articles not reporting relevant clinical, biological, or molecular outcomes.
- Non-peer-reviewed articles.
- Studies published in languages other than English.

## Chemistry and pharmacokinetics

**Fig. 1:**
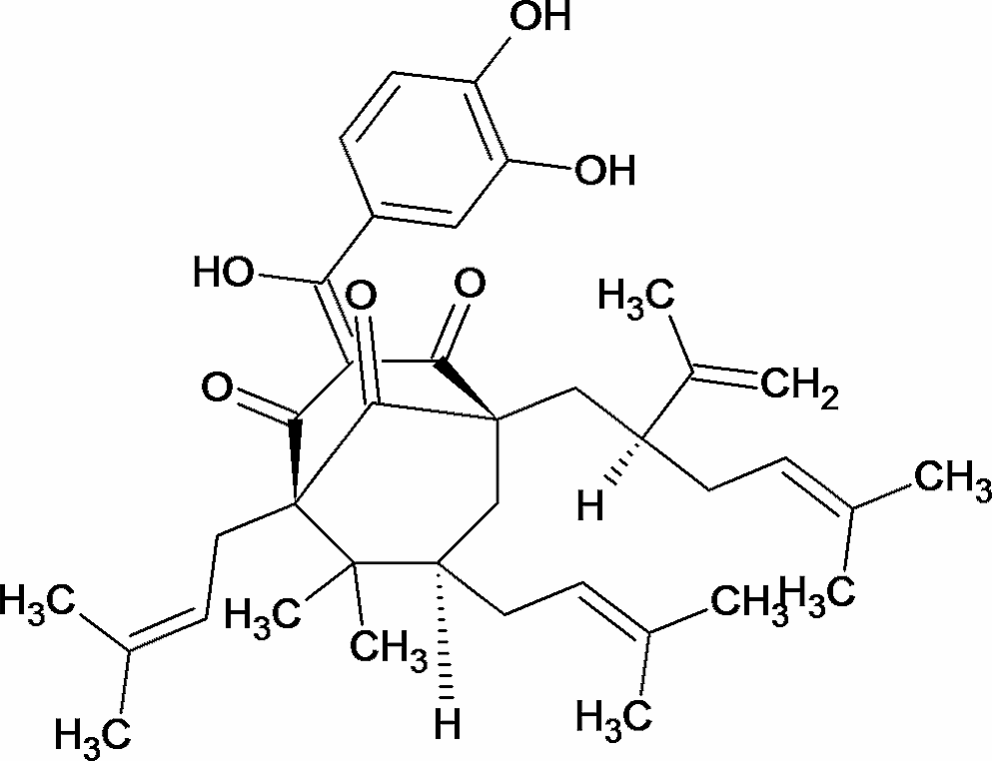
Chemical structure of polyisoprenylated benzophenone (Garcinol)

In a chemical way Garcinol, a polyisoprenylated benzophenone (Fig. 1), is naturally occurring phytochemical found in the plants *Garcinia cambogia*, also known as Malabar tamarind, and *Garcinia indica*, which belong to the Clusiaceae family and are commonly known as Kokum (Fernando et al. 2019). In current methods of methanol / and ethyl acetate extraction using chopped dried kokum plums produces good yields of garcinol (up to 5 g from 500 g dried kokum plums). Subsequently, yellow needles like crystal with a melting point of 132 °C are produced by crystallizing the concentrated eluate of hexane fraction through column chromatography of the concentrated ethyl acetate extract (silica gel 60, ethyl acetate/hexane 1:2) (Schobert and Biersack 2019; Ullah and Ahmad 2016). Another effective large-scale isolation technique was developed by washing the *Garcinia indica* fruits with water to remove the hydroxycitric acid and extracted using methanol. The methanol extract loaded on Celite was eluted using hexane. Finally, the extract of hexane was separated using column chromatography (Schobert and Biersack 2019).

Chemists have also recently extracted garcinol from the fruits of *Garcinia multiflora*, a South Chinese medicinal plant (Hemshekhar et al. 2011; Liu et al. 2017). 500 mg of garcinol were extracted from 5.2 kg of dried *G. multiflora* fruits. Garcinol was obtained by extracting the powdered dried fruits of *G. multiflora* using 95% ethanol and petroleum ether using column chromatography on silica gel followed by recrystallization (Liu et al. 2017). Additionally, plants belonging to the *Garcinia* species *G. morella*,* G. yunnanensis*,* G. xanthochymus*, and *G. travancorica* were shown to contain and yield *Garcinia* ethanol (Choudhury et al. 2018; Zheng et al. 2017; Jackson et al. 2015; Anu Aravind et al. 2016). Furthermore, it was recently discovered that the stem bark of the *Garcinia* species, *G. buchananii*, has also garcinol presence (Stark et al. 2015).

## Safety aspects

*Garcinia indica* is the source of Garcinol. Assessing its safety is essential for therapeutic applications (Lim et al. 2021). Various areas of safety were examined in studies. These comprise the following: potential against inflammation, potential against cancer, reproductive toxicity, developmental toxicity, acute toxicity, and repeated dosage toxicity (Majeed et al. 2018). The acute and sub-chronic toxicity of 40% standardized garcinol was evaluated in rodents. This research had OECD criteria in mind. The toxicity of carcinol to rodents was found to be minimal. On clinical symptoms, behavior, development, reproduction, or histology, no negative consequences were observed. The hazardous nature of semisynthetic derivatives of garcinol (Beerwala et al. 2024). In a preclinical model, they investigated the metastasis of pulmonary melanoma. In mice in good health, the compounds had a beneficial effect without generating systemic damage. The anti-cancer and anti-inflammatory properties of Garcinol safety (Liu et al. 2015). They looked into how the inflammatory Aquaporins are modulated by Garcinol. It successfully inhibited the generation of proinflammatory cytokines in cell culture models. Garcinol inhibits alpha-amylase, indicating that *Garcinia indica* extract may have promise as an antidiabetic drug, according to research conducted in vitro and in silico (Kalita and Das 2024).

Preclinical research has shown encouraging results, however, the toxicity profile of Garcinol is still unknown to experts. Its safety for clinical application has to be further investigated (Majeed et al. 2018). Garcinol has an excellent safety profile according to preclinical research, and animal experiments have shown no significant harm. Nevertheless, more investigation is required by scientists to completely comprehend the clinical safety and treatment potential of ganciclovir for inflammatory and cancerous diseases. This includes pharmacokinetic investigations and clinical trials (Butnariu et al. 2022).

## Major gastrointestinal cancer

### Esophageal cancer

**Fig. 2:**
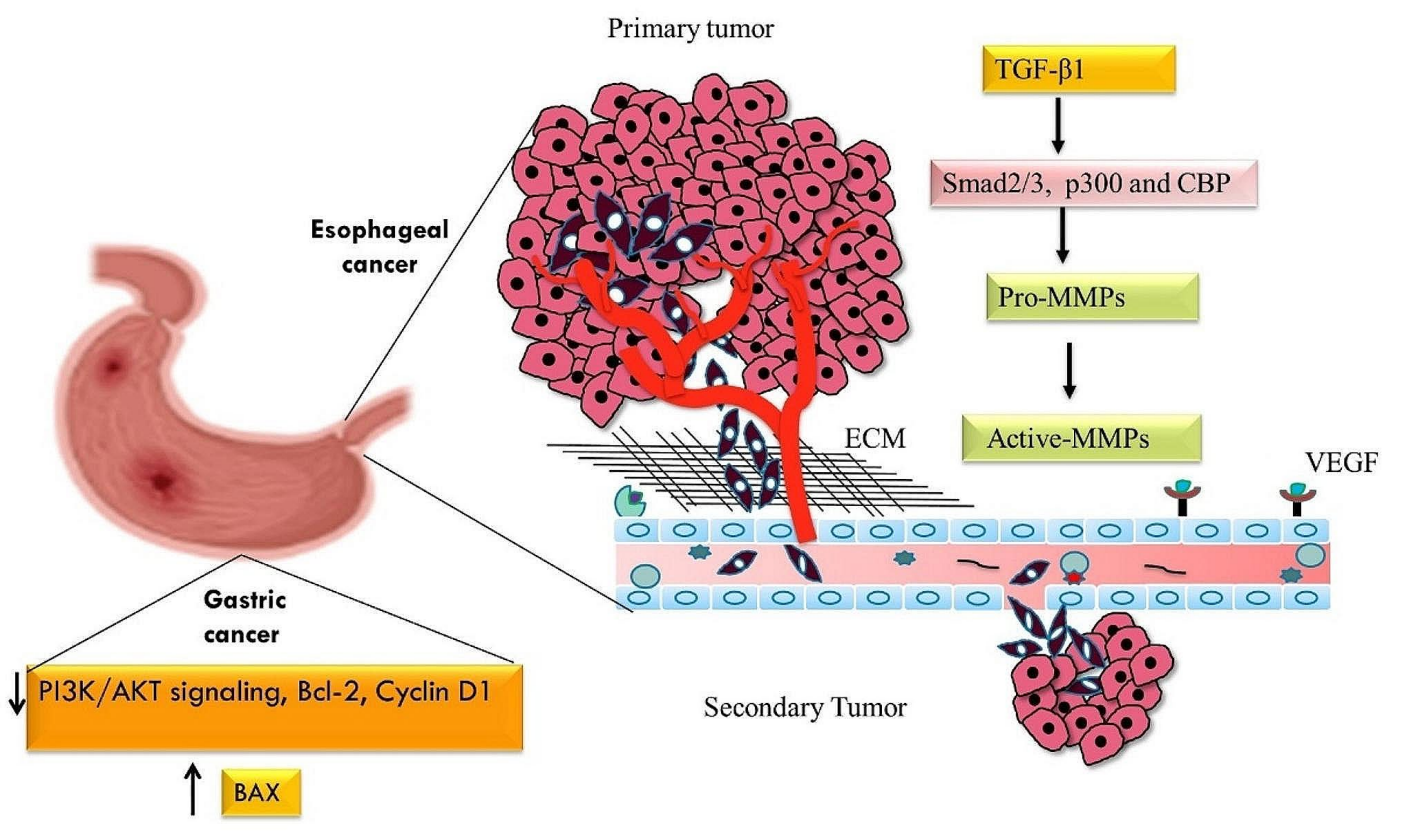
Illustration of the Cancer Pathway in the Stomach and Esophagus: This detailed diagram highlights the molecular mechanisms and key factors involved in the development and progression of cancer within the gastrointestinal tract. The pathway underscores critical mutations, signaling pathways, and cellular changes that contribute to the onset and metastasis of stomach and esophageal cancers

Esophageal cancer emerges as a significant challenge within the landscape of global health, as revealed by the GLOBOCAN 2018 data. This cancer ranked as the eighth most prevalent cancer worldwide and stands as the sixth most common cause of mortality from cancer, its effect is profoundly felt overpopulation. This cancer takes a terrible toll every year, taking the lives of more than 500,000 people worldwide—roughly 5.3% of all cancer-related deaths. Its range is extensive and significant global variation in distribution has been observed. In addition, the five-year survival rate is consistently poor, ranging from 15 to 20%. (Then et al. 2020; Uhlenhopp et al. 2020; Abbas and Krasna 2017). A study conducted on human esophageal cancer cell lines KYSE150 andgra KYSE450 suggests that Garcinol exerts significant inhibitory effects on metastasis-related pathways. Through its inhibition of p300 and CBP activity, garcinol interferes with the transcriptional machinery necessary for the production of metastasis-related genes, leads to inhibition of the transition of cancer cells to a more invasive phenotype. Moreover, Garcinol suppresses TGF-β1-induced activation of Smad2/3 and p300, attenuating the transcriptional activation of EMT markers (Fig. 2). Animal studies further support Garcinol’s potential as a therapeutic agent by demonstrating its ability to inhibit pulmonary metastasis in esophageal cancer models (Wang et al. 2020).

### Gastric (stomach) cancer

Gastric cancer ranks as the fifth most common cancer worldwide, it stands as the fourth most common cause of cancer-related deaths, with nearly 989,600 new cases reported in 2008 and accounts for over 738,000 deaths worldwide each year. It should be noted that there is a significant gender difference in the incidence of gastric cancer, as men are twice as likely as women to develop this cancer (Gallanis et al. 2024; Sitarz et al. 2018). A research conducted by Zheng et al. on the human gastric cancer cell line HGC-27, shows that garcinol exhibits multiple approaches in combating gastric cancer. It reduces the expression of Cyclin D1, which slows down the rate of cell division and suppresses cell proliferation. Garcinol also inhibits invasion and metastasis through the downregulation of matrix metalloproteinases (MMPs), very crucial enzymes that break down the extracellular matrix. In addition, garcinol induces programmed cell death (apoptosis) in gastric cancer cells by regulating the important balance between pro- and anti-apoptotic proteins. It downregulates the anti-apoptotic protein Bcl-2 (B-cell lymphoma) whereas upregulating the pro-apoptotic protein BAX (Bcl X-related protein 2), eventually triggers the apoptotic pathway (Fig. 2). Garcinol also modulate the PI3K/AKT signaling pathway, which is often dysregulated in gastric cancer. By inhibiting AKT (Protein Kinase B) activation and downstream signaling, garcinol effectively suppresses neoplastic transformation of gastric cells. These finding together highlight the excellent potential of the therapeutic drug garcinol in the treatment of gastric cancer and provide an attractive avenue for further research and clinical development (Zheng et al. 2020).

### Gallbladder cancer

Gallbladder cancer (GBC) is a malignancy of the biliary tract, and it accounts for 1.2% of global cancer diagnoses but 1.7% of cancer deaths, according to GLOBOCAN 2018. Despite the gallbladder’s small size, GBC causes around 165,000 deaths annually, making it the 22nd most incident but 17th most deadly cancer worldwide. It is more common among women than men, with 2018 estimates of 122,000 cases in women versus 97,000 in men, may be due to women’s longer life expectancy. GBC is very deadly due to its late diagnosis, in the US only 20% of cases are detected early, with advanced-stage median survival around a year (Rawla et al. 2019b). This cancer is more common in certain ethnicities and regions, notably the Indo-Gangetic plains of India, Mapuche Indians in Chile, and parts of South America. India, particularly its northern, northeastern, central, and eastern regions, accounts for about 10% of the global GBC cases (Dutta et al. 2019). A study conducted on gallbladder cancer (GBC) cell lines, GBC-SD and NOZ lines by Duan et al., revealed garcinol’s significant therapeutic benefits. The study found that garcinol inhibited the development and invasion of these cancer cells in a dose- and time-dependent manner. Garcinol inhibits the activity of matrix metalloproteinase 2 (MMP2) and MMP9 by downregulating their mRNA levels, and further inhibits the activation of the Stat3 and Akt signaling pathways in GBC-SD cells (Duan et al. 2018).

### Pancreatic cancer

**Fig. 3:**
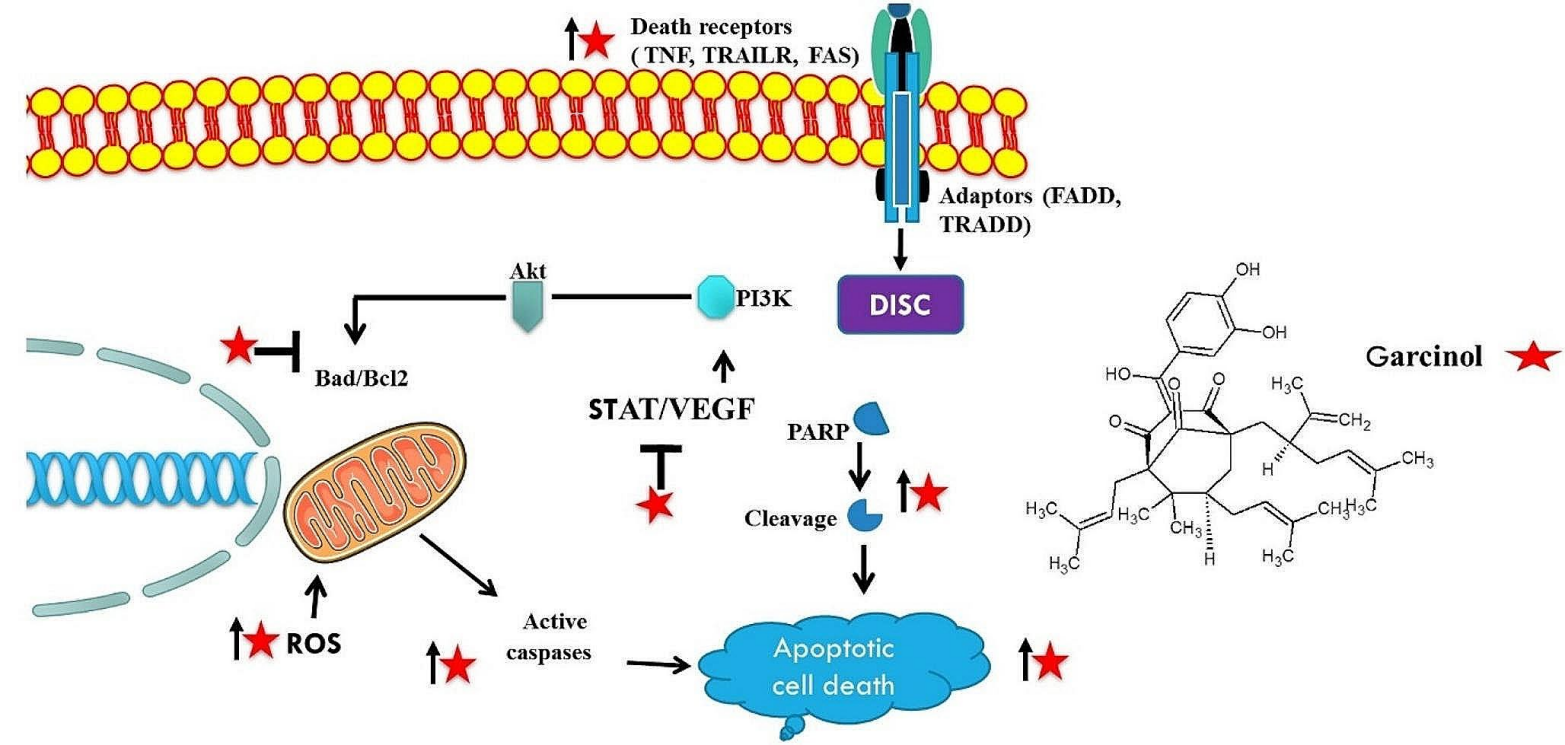
Garcinol promotes apoptosis in liver cancer cells through both intrinsic and extrinsic pathways. In the extrinsic pathway, it upregulates death receptors (such as Fas ligand, TNF, and TRAIL), sensitizes cells to death ligands (via FADD and TRADD), and facilitates the formation and activation of the DISC (Death-Inducing Signaling Complex). This cascade ultimately triggers caspases, leading to apoptosis. In the intrinsic pathway, Garcinol activates caspases that cleave PARP(Poly (ADP-ribose) polymerase), a protein critical for DNA repair and cell death processes. It also boosts pro-apoptotic proteins like Bax, reduces anti-apoptotic proteins like Bcl-2, induces oxidative stress through increased ROS production, and suppresses STAT3 activation, further decreasing the transcription of genes that support cell survival and results in apoptosis

Pancreatic cancer stands out as a strong foe in the global health landscape, as it is the seventh greatest cause of cancer-related deaths globally and ranks 14th among the most common malignancies. Globocan data 2018, reported 0.43 million fatalities 0.46 million new cases and 0.43 million fatalities from this cancer. It frequently affects the elderly, with an incredible 90% of newly diagnosed patients being above the age of 55, with the majority falling between the ages of 70 and 80. Compared to women (4.0 per 100,000, 215,885 instances), men are more likely to experience it (5.5 per 100,000, 243,033 cases) (Rawla et al. 2019a). In India, pancreatic cancer ranks 24th with 10,860 new cases (1.03%) and 18th in mortality (McGuigan et al. 2018; Zhao and Liu 2020; Gaidhani and Balasubramaniam 2021; Rawla et al. 2019b). A comprehensive study conducted on pancreatic cancer cell lines (especially BxPC-3 and Panc-1) has elucidated the multifaceted role of garcinol in inhibiting various molecular pathways associated with pancreatic cancer progression. Western immunoblotting experiments conducted by Parasramka and colleagues revealed that Garcinol effectively inhibits molecules such as X-linked inhibitor of apoptosis (X-IAP) and cellular inhibitor of apoptosis (cIAP), which block apoptosis in pancreatic cancer cells by inhibiting caspases (caspase 3 and caspase 9), crucial enzymes in apoptosis. Garcinol reduces the production of prostaglandin E2 (PGE2), a pro-inflammatory molecule, by downregulating cyclooxygenase-2 (COX-2), an enzyme involved in PGE2 synthesis (Saadat and Gupta 2012). This action is mediated through the inhibition of NF-κB binding activity, as concluded by Electrophoretic Mobility Shift Assay (EMSA). NF-κB is a transcription factor that plays a crucial role in the survival and proliferation of pancreatic cancer cells (Bao at al. 2011). Garcinol inhibits matrix metalloproteinase-9 (MMP-9), an enzyme involved in tumor cell invasion, and vascular endothelial growth factor (VEGF), a key regulator of angiogenesis (the formation of new blood vessels). Additionally, garcinol suppresses the secretion of interleukin-8 (IL-8), a pro-inflammatory cytokine implicated in tumor angiogenesis, invasiveness, and metastasis (Parasramka and Gupta 2011). The study conducted on a mouse model demonstrated that dietary Garcinol significantly improved the survival rate of KPC mice. Notably, when administered alone, Garcinol exhibited the capability to arrest tumor progression and reduce the size of certain tumors, as assessed by MRI and ultrasound imaging (Saadat 2013). Further investigation conducted by Pandita et al. on the combined application of dietary compounds, garcinol, and curcumin, along with the study by Parasramka et al. on garcinol along with gemcitabine, shows a synergistic potential in suppressing the growth of human pancreatic cancer (PaCa) cells (Fig. 3). This integrated result presents a promising approach for treatment, potentially offering a more effective therapeutic option compared to the utilization of either compound alone (Pandita et al. 2014; Parasramka and Gupta 2012).

### Liver cancer

Based on 2020 statistics, liver cancer stands as the sixth most prevalent cancer worldwide and ranks third in terms of cancer-related fatalities, with approximately 905,700 individuals diagnosed and 830,200 deaths from the cancer. In particular, the incidence in regions such as sub-Saharan Africa and Southeast Asia is significantly higher than in the United States. The fifth most prevalent cancer in males and the seventh most common in women is liver cancer. (Rumgay et al., 2022; Sung et al. 2021; Ferlay et al. 2015). Various studies have been done till date to find progression and cure of this cancer. The studies conducted by Sethi et al. they investigated the role of garcinol in liver cancer treatment, employing a comprehensive approach that included in vitro experiments using liver cancer cells C3A and HUH-7, in vivo studies with a mouse model, and computational analysis. Their findings revealed promising results, suggesting that garcinol targets the STAT3 signaling pathway, a key regulator of cancer progression. By inhibiting STAT3 phosphorylation, garcinol disrupts STAT3 dimerization, leading to the downregulation of critical STAT3-regulated genes involved in cancer cell survival and proliferation, such as cyclin D1, Bcl-2, Bcl-xL, survivin, Mcl-1, and VEGF. This multifaceted action not only hampers tumor growth but also induces apoptotic cell death, as evidenced by the activation of pro-caspase-3 and cleavage of PARP. Additionally, garcinol exhibits anti-angiogenic effects by targeting VEGF expression. Furthermore, its inhibition of STAT3 acetylation impedes the nuclear localization and DNA binding of STAT3, further reducing its transcriptional activity and contributing to its anti-cancer effects. Computational modeling supports these findings by suggesting direct binding of garcinol to the SH2 domain of STAT3, hindering its dimerization and transcriptional activity. In preclinical studies using a nude mouse model, garcinol significantly suppresses HCC tumor growth by suppressing expression of p-STAT3 in garcinol treated tumor tissues (Sethi et al. 2014). One more study conducted by Cheng’s et al. on Hep3B cells underscores garcinol’s role in inducing ROS production, triggering oxidative stress and apoptotic cell death. Furthermore, garcinol promotes TRAIL-induced apoptosis by modifying death receptors and anti-apoptotic proteins, as well as promoting GADD153 expression, which amplifies apoptotic events downstream (Cheng et al. 2010). Together, these findings indicate garcinol as a viable therapeutic agent against liver cancer, with several routes for intervention due to its unique modes of action.

### Colorectal cancer

**Fig. 4:**
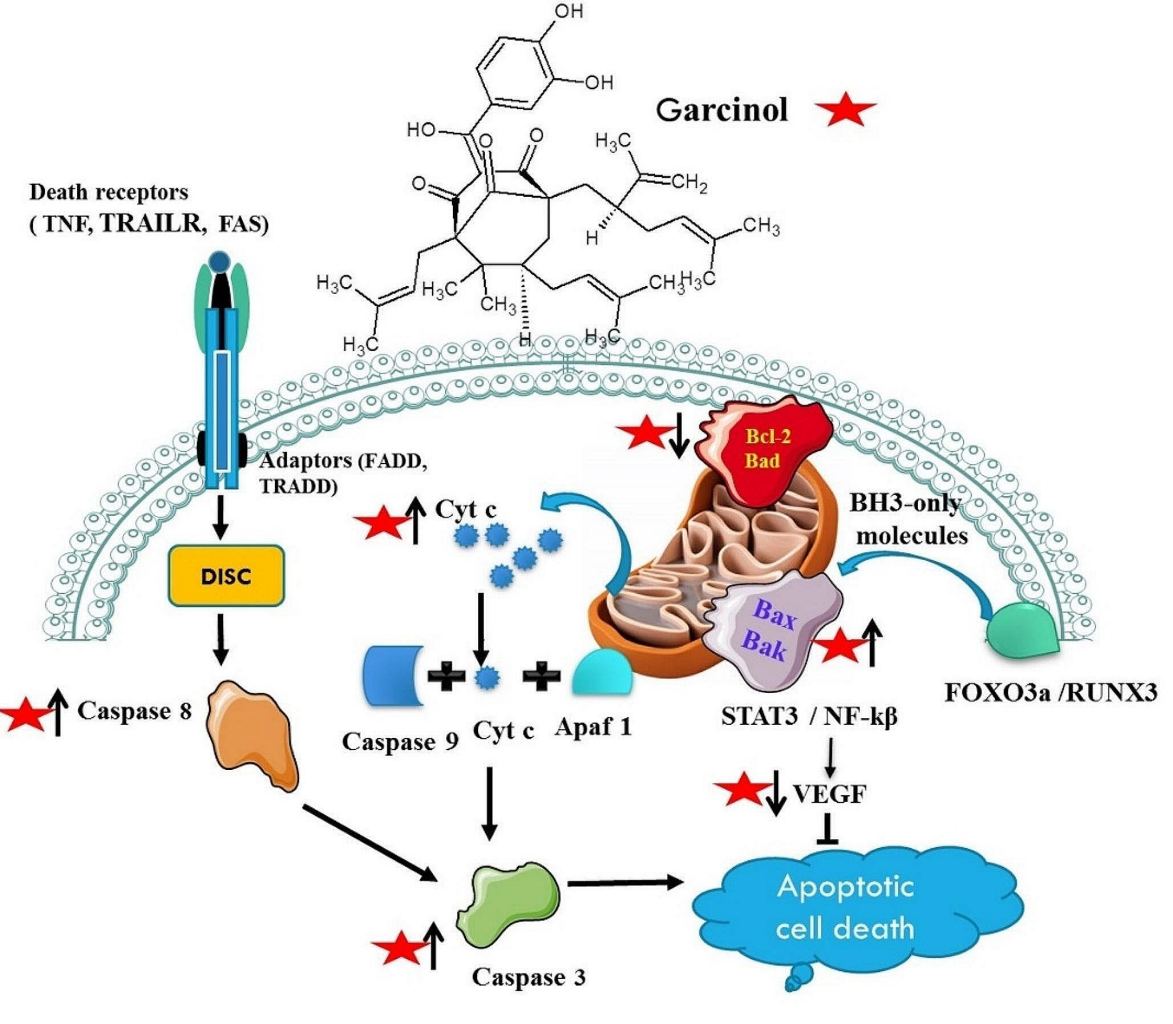
Mechanism of Garcinol function- Garcinol triggers apoptosis by the inhibition of STAT3 and NF-κB pathways (anti-apoptotic pathways), and downregulates anti-apoptotic protein Bcl-2 and, upregulating Bax which undergoes a conformational change and translocate to the mitochondria, and oligomerizes to form pores in the mitochondrial outer membrane which cause the release of cytochrome c into the cytosol. Cytochrome c binds to Apaf-1 (apoptotic protease activating factor-1) and procaspase-9 to form the apoptosome, leading to the activation of caspase-9. Which in turn activates executioner caspases 3 and results in apoptosis

Globally, colorectal cancer (CRC) is the third most common cancer diagnosis and the fourth most common cause of cancer-related mortality, there were almost 700,000 fatalities and 1.4 million new instances of colorectal cancer diagnosed in 2012 alone. Above data highlight its serious impact on public health and highlight the pressing need for efficient methods of early detection, treatment, and prevention of cancer (Arnold et al. 2017; Mármol et al. 2017; Sawicki et al. 2021). The investigation carried out on HT-29 colorectal cancer cells reveals the complex mechanisms by which garcinol demonstrates its powerful anti-cancer properties, providing deep understanding into its potential as therapeutic drug. Study suggest that garcinol inhibits FAK auto-phosphorylation on Tyr397 in a dose-dependent manner, triggering a series of molecular events that ultimately lead to the induction of apoptosis. Furthermore, garcinol exhibits notable suppression of key signaling pathways including Ras/MAPK, PI3K/Akt, and Src, which are essential controllers of cell division, proliferation, and survival. Another study suggests that, garcinol triggers mitochondrial disturbance, resulting in the release of cytochrome c into the cytosol. The subsequent activation of caspase-3, which is dependent on caspase-9, coordinates the amplification of apoptosis. This process is further enhanced by the cleavage of PARP, a crucial event in programmed cell death (Fig. 4). Garcinol promotes pro-apoptotic signaling by reducing the levels of the anti-apoptotic Bcl-2 protein and increasing the levels of the pro-apoptotic Bax protein. As a result, garcinol intensifies the induction of apoptosis in cancer cells. Garcinol not only affects apoptosis but it also acts as a potent inhibitor of colorectal cancer progression by targeting various key molecular players It downregulates various important genes such as mPGES-1, CXCR4, and VEGF, and reduces cancer aggressiveness. Moreover, garcinol shows a remarkable ability to prevent the invasive potential of HT-29 cells by down-regulating matrix metalloproteinase (MMP7) protein levels, as evidenced by real-time PCR analysis revealed significant decreases in MMP-2 and MMP-9 expression (Liao et al. 2005; Ranjbarnejad et al. 2017).

## Role of nanotechnology and synergism

Nanotechnology makes the delivery of Garcinol more effective in the battle against gastrointestinal malignancies. Garcinol, an anti-cancer bio-chemical for GI cancers (Table 1), is derived from *Garcinia indica*. However, Garcinol has difficulties getting to tumor effectively. Nanoparticles engineered for targeted delivery of specific compound (specially for tumor microenvironment remodelling) have emerged as promising tools in cancer treatment (Lu et al. 2024). Garcinol is wrapped with nanoparticles, which enhance its solubility and target tumor locations (Jacob et al. 2021). In combination with other anti-cancer agents, garcinol produces a synergistic effect. Nanoparticles enable controlled release with specific targeting of garcinol, further potentiating its effect on cancer cells (Aggarwal et al. 2020). Controlled release of Garcinol, specifically in the colon, is made possible by using pH-responsive nanoparticles. This minimizes side effects across the body and offers targeted treatment for gastrointestinal tumors (Brito et al. 2022). As a result, cancer cells can be actively targeted, improving the specificity and effectiveness of treatments. For better use, Garcinol may be encapsulated in nanoparticles which can shield it from the body’s breakdown and make it easier to absorb by the body (Li et al. 2023). Combine effect of peptides and nanoparticles enhance their ability to target specifically tumor cells (Dai et al. 2024). Exosomes are small, membrane-bound vesicles released from cells into the extracellular environment. As carriers of anti-tumor agents, they can modify the tumor microenvironment, results in significant change in cancer cell proliferation and migration (Paskeh et al. 2022). We have also looked into possible synergistic benefits among garcinol and other phytochemicals, like curcumin, along with garcinol and the cancer drugs currently available for treatments in pancreatic cancer cell lines, such as gemcitabine (Kopytko et al. 2021). Using a combinatorial approach, the findings show that garcinol and curcumin suppress Pancreatic cancer cells, Panc 1 and BxPC3, more so than either drug alone by increasing the induction of apoptosis (Rizvi et al., 2024). Using different doses and incubating periods, garcinol and gemcitabine were given alone and combined with human pancreatic cancer cell lines, BxPC 3 and Panc-1, that contained the wild-type and mutant K-ras genotypes, respectively (Mane et al. 2012). This treatment is intended to observe the level of apoptotic cell death and growth reduction. Compared to the individual therapies, the combination of garcinol and gemcitabine demonstrated a significant decrease in cell proliferation and an increase in apoptosis. Additionally, garcinol downregulated NF-κB, VEGF, Il 8, MMP-9, and activated PARP cleavage in concert with gemcitabine to promote its activity (Zheng et al. 2020). This highlights garcinol’s potential greater in combinatorial treatment approaches. However, its clinical translation faces several challenges. Among the most significant is their poor bioavailability which could be improved by their solubility and targeted delivery enhancement through nanotechnology (Ahmad et al. 2011). In addition, because interactions between various chemopreventive agents can produce different results due to their diversity, achieving the combined effect is still difficult. Understanding and optimizing these interactions requires extensive preclinical and clinical studies (Hong et al. 2007).

**Table 1:** Effect of Garcinol on different types of cancer

S.no	Type of Cancer	Subjective model	Physiological effect	Mechanism of action	References
1.	Esophageal cancer	KYSE150 and KYSE450, mouse model	Anti-metastasis	↓ p300/CBP and p-Smad2/3, ↑ HAT, ↓TGF-β, ↓Ki-67	Wang et al. 2020; Staebler et al. 2024
2.	Gastric cancer	HGC-27 cell line	Apoptosis induction and Anti-proliferative effect	↓ PI3K/AKT and AKTp‑Thr308 and AKTp‑ser473, ↓ MMP-2 and MMP-9	Zheng et al. 2020; Rizvi et al., 2024
3.	Colon cancer	HT-29 cell line	Apoptosis induction	↓MMP-7, ↓ FAK, ↑Src, ↓ PI3K, ↑Caspase-3	Liao et al. 2005
4.	Hepatocellular carcinoma	Hep3B cells	Apoptosis induction	↑GADD153, ↑Bax/Bcl-2, ↑Caspase-8 and tBid, ↑caspase-3 and caspase-9, ↑DFF-45	Cheng et al. 2010
5.	Pancreatic cancer	BxPC-3 and Panc-1 cell lines	Apoptosis induction, Antiproliferative Effect	↓ MMP-9, ↓ IL-8, ↓ PGE-2, and ↓ VEGF	Parasramka, & Gupta, 2011; Zhu et al. 2024
6.	Colorectal cancer	HT-29 cell lines	Antiproliferative Effect	↓ mPGES-1, ↓HIF-1α, ↓VEGF, ↓CXCR4, ↓MMP-2, and MMP-9, ↑caspase 3	Ranjbarnejad et al. 2017; Basumatary et al. 2024
7.	Colon cancer	HT-29 and HCT-116 cell lines	Antiproliferative Effect, Apoptosis induction	↑AKT, ↑survivin, ↑ERK	Hong et al. 2007; Unnikrishnan Meenakshi et al. 2024
8.	Hepatocellular carcinoma	MH1C1 and HepG2 cells	Apoptosis induction and Anti proliferative effect, Cell cycle arrest	↓cyclin E2, ↓bcl-2 and ↓cyclin B1, ↑14-3-3 σ	Ohnishi et al. 2004
9.	Hepatocellular carcinoma	HCC cell lines	Antimetasis, Apoptosis induction	↓STAT3, ↓ D1, ↓Bcl-2, ↓Bcl-xL, ↓Mcl-1, ↓survivin, and ↓VEGF	Sethi et al. 2014; Rao Gajula et al. 2023

## Conclusion and future perspectives

The evidence presented highlights the potential of garcinol as a promising therapeutic agent against gastrointestinal cancers. Studies demonstrate its ability to inhibit cancer cell proliferation, induce apoptosis, and suppress metastasis through various molecular mechanisms. Moreover, its low toxicity and high patient acceptance rate make it an attractive candidate for cancer treatment. It enhances chemotherapy effectiveness, exhibits anti-inflammatory effects, inhibits angiogenesis, and has a favorable safety profile in preclinical studies. These multi-targeted actions suggest Garcinol’s potential in both therapeutic and preventive oncology (Behera et al. 2016). However, challenges such as poor bioavailability and delivery issues need to be addressed. Future research should focus on optimizing garcinol’s delivery, possibly through nanotechnology, to enhance its efficacy and minimize side effects. Additionally, investigating synergistic combinations with other phytochemicals or conventional cancer drugs could lead to more effective treatments. Clinical trials are needed to validate garcinol’s efficacy and safety in patients. Additionally, further exploration of garcinol’s mechanisms of action and its effects on different types and stages of gastrointestinal cancers would provide valuable insights.

## Data Availability

No datasets were generated or analysed during the current study.

## References

[CR1] Abbas G, Krasna M (2017) Overview of esophageal cancer. Annals Cardiothorac Surg 6(2):131 10.21037/acs.2017.03.03 PMC5387155 28447001

[CR2] Aggarwal V, Tuli HS, Kaur J, Aggarwal D, Parashar G, Chaturvedi Parashar N, Ahn KS (2020) Garcinol exhibits anti-neoplastic effects by targeting diverse oncogenic factors in tumor cells. Biomedicines 8(5):103 32365899 10.3390/biomedicines8050103 PMC7277375

[CR3] Ahmad A, Padhye S, Sarkar FH (2011) Role of novel nutraceuticals garcinol, plumbagin and mangiferin in the prevention and therapy of human malignancies: mechanisms of anticancer activity. Nutraceuticals and Cancer. Springer Netherlands, Dordrecht, pp 179–199

[CR4] Anu Aravind AP, Asha KRT, Rameshkumar KB (2016) Phytochemical analysis and antioxidant potential of the leaves of Garcinia Travancorica Bedd. Nat Prod Res 30(2):232–236 25982126 10.1080/14786419.2015.1043551

[CR5] Arnold M, Sierra MS, Laversanne M, Soerjomataram I, Jemal A, Bray F (2017) Global patterns and trends in colorectal cancer incidence and mortality. Gut 66(4):683–691 26818619 10.1136/gutjnl-2015-310912

[CR6] Asano T (2020) Drug resistance in cancer therapy and the role of epigenetics. J Nippon Med School 87(5):244–251 10.1272/jnms.JNMS.2020_87-508 32475898

[CR7] Bao B, Padhye S, Sarkar FH (2011) Garcinol-induced apoptosis in prostate and pancreatic cancer cells is mediated by NF-κB signaling Aamir Ahmad1, Zhiwei Wang1, Christine Wojewoda1, Raza Ali1, Dejuan Kong1, Ma’in Y. Maitah1, Sanjeev Banerjee1. Front Biosci 3:1483–1492 10.2741/e349 21622152

[CR8] Basumatary D, Kashyap B, Baruah P, Borah JC (2024) Prophylactic function of Garcinia Morella dried fruits in preventing intestinal barrier disruption by lowering tight junction permeability and inflammation. Food Bioscience 57:103408 10.1016/j.fbio.2023.103408

[CR9] Beerwala FA, Kolambkar SV, Patil VS, Darasaguppe HR, Khatib NA, Bhandare VV, Roy S (2024) Decoding the alpha-amylase inhibitory activity of Garcinia indica Choisy by computational and experimental studies. South Afr J Bot 165:14–29 10.1016/j.sajb.2023.12.014

[CR10] Behera AK, Swamy MM, Natesh N, Kundu TK (2016) Garcinol and its role in chronic diseases. Anti-inflammatory Nutraceuticals and Chronic Diseases, pp 435–452 10.1007/978-3-319-41334-1_18 27671827

[CR11] Brito LDC, Marques AM, da Cunha Camillo F, Figueiredo MR (2022) Garcinia Spp: products and by-products with potential pharmacological application in cancer. Food Bioscience 50:102110 10.1016/j.fbio.2022.102110

[CR12] Butnariu M, Quispe C, Sharifi-Ra J, Pons-Fuste E, Lopez-Jorne P, Zam W, Chen JT (2022) Naturally-occurring bioactives in oral cancer: preclinical and clinical studies, bottlenecks and future directions 10.31083/j.fbs1403024 36137983

[CR13] Cao W, Chen HD, Yu YW, Li N, Chen WQ (2021) Changing profiles of cancer burden worldwide and in China: a secondary analysis of the global cancer statistics 2020. Chin Med J 134(07):783–791 33734139 10.1097/CM9.0000000000001474 PMC8104205

[CR14] Cheng AC, Tsai ML, Liu CM, Lee MF, Nagabhushanam K, Ho CT, Pan MH (2010) Garcinol inhibits cell growth in hepatocellular carcinoma Hep3B cells through induction of ROS-dependent apoptosis. Food Funct 1(3):301–307 21776480 10.1039/c0fo00134a

[CR15] Choudhury B, Kandimalla R, Elancheran R, Bharali R, Kotoky J (2018) Garcinia Morella fruit, a promising source of antioxidant and anti-inflammatory agents induces breast cancer cell death via triggering apoptotic pathway. Biomed Pharmacother 103:562–573 29677543 10.1016/j.biopha.2018.04.068

[CR16] Dai J, Ashrafizadeh M, Aref AR, Sethi G, Ertas YN (2024) Peptide-functionalized, -assembled and -loaded nanoparticles in cancer therapy. Drug Discovery Today 103981. 10.1016/j.drudis.2024.103981 10.1016/j.drudis.2024.103981 38614161

[CR17] Duan YT, Yang XA, Fang LY, Wang JH, Liu Q (2018) Anti-proliferative and anti-invasive effects of garcinol from Garcinia indica on gallbladder carcinoma cells. Die Pharmazie-An Int J Pharm Sci 73(7):413–417. 10.1691/ph.2018.8366 10.1691/ph.2018.8366 30001777

[CR19] Dutta U, Bush N, Kalsi D, Popli P, Kapoor VK (2019) Epidemiology of gallbladder cancer in India. Chin Clin Oncol 8(4):33–33. 10.21037/cco.2019.08.03 31484488 10.21037/cco.2019.08.03

[CR18] Dutta S, Ganguly A, Chatterjee K, Spada S, Mukherjee S (2023) Targets of immune escape mechanisms in cancer: basis for development and evolution of cancer immune checkpoint inhibitors. Biology 12(2):218 36829496 10.3390/biology12020218 PMC9952779

[CR20] Ferlay J, Soerjomataram I, Dikshit R, Eser S, Mathers C, Rebelo M, Bray F (2015) Cancer incidence and mortality worldwide: sources, methods and major patterns in GLOBOCAN 2012. Int J Cancer 136(5):E359–E386 25220842 10.1002/ijc.29210

[CR21] Fernando HN, Kumarasinghe UR, Gunasekara CP, Wijekoon SK, Ekanayaka AK, Rajapaksha SP, Jayaweera PM (2019) Synthesis, characterization and antimicrobial activity of garcinol capped silver nanoparticles 10.4014/jmb.1904.04032 31387343

[CR22] Gaidhani RH, Balasubramaniam G (2021) An epidemiological review of pancreatic cancer with special reference to India. Indian J Med Sci 73(1):99–109 10.25259/IJMS_92_2020

[CR23] Gallanis AF, Mannes AJ, Davis JL (2024) Gastric Cancer surgery. Anesthesia for oncological surgery. Springer International Publishing, Cham, pp 257–261

[CR24] Hemshekhar M, Sunitha K, Santhosh MS, Devaraja S, Kemparaju K, Vishwanath BS, Girish KS (2011) An overview on genus Garcinia: phytochemical and therapeutical aspects. Phytochem Rev 10:325–351 10.1007/s11101-011-9207-3

[CR25] Hong J, Kwon SJ, Sang S, Ju J, Zhou JN, Ho CT, Yang CS (2007) Effects of garcinol and its derivatives on intestinal cell growth: inhibitory effects and autoxidation-dependent growth-stimulatory effects. Free Radic Biol Med 42(8):1211–1221 17382202 10.1016/j.freeradbiomed.2007.01.016

[CR26] Jackson DN, Yang L, Wu S, Kennelly EJ, Lipke PN (2015) Garcinia xanthochymus benzophenones promote hyphal apoptosis and potentiate activity of fluconazole against Candida albicans biofilms. Antimicrob Agents Chemother 59(10):6032–6038 26195512 10.1128/AAC.00820-15 PMC4576022

[CR27] Jacob EM, Borah A, Pillai SC, Kumar DS (2021) Garcinol encapsulated pH-sensitive biodegradable nanoparticles: a novel therapeutic strategy for the treatment of inflammatory bowel disease. Polymers 13(6):862 33799680 10.3390/polym13060862 PMC7999919

[CR28] Kalita A, Das M (2024) Aquaporins (AQPs) as a marker in the physiology of inflammation and its interaction studies with garcinol. Inflammopharmacology, pp 1–18 10.1007/s10787-023-01412-9 38267609

[CR29] Kallingal A, Olszewski M, Maciejewska N, Brankiewicz W, Baginski M (2023) Cancer immune escape: the role of antigen presentation machinery. J Cancer Res Clin Oncol 149(10):8131–8141 37031434 10.1007/s00432-023-04737-8 PMC10374767

[CR31] Kaur R, Bhardwaj A, Gupta S (2023) Cancer treatment therapies: traditional to modern approaches to combat cancers. Mol Biol Rep 50(11):9663–9676 37828275 10.1007/s11033-023-08809-3

[CR32] Kim SK, Cho SW (2022) The evasion mechanisms of cancer immunity and drug intervention in the tumor microenvironment. Front Pharmacol 13:868695 35685630 10.3389/fphar.2022.868695 PMC9171538

[CR33] Kopytko P, Piotrowska K, Janisiak J, Tarnowski M (2021) Garcinol—a natural histone acetyltransferase inhibitor and new anti-cancer epigenetic drug. Int J Mol Sci 22(6):2828 33799504 10.3390/ijms22062828 PMC8001519

[CR34] Li J, Ye F, Xu X, Xu P, Wang P, Zheng G, Shen H (2023) Targeting macrophage M1 polarization suppression through PCAF inhibition alleviates autoimmune arthritis via synergistic NF-κB and H3K9Ac blockade. J Nanobiotechnol 21(1):280 10.1186/s12951-023-02012-z PMC10439630 37598147

[CR35] Liao CH, Sang S, Ho CT, Lin JK (2005) Garcinol modulates tyrosine phosphorylation of FAK and subsequently induces apoptosis through down-regulation of src, ERK, and akt survival signaling in human colon cancer cells. J Cell Biochem 96(1):155–169 16052481 10.1002/jcb.20540

[CR36] Liberti MV, Locasale JW (2016) The Warburg effect: how does it benefit cancer cells? Trends Biochem Sci 41(3):211–218 26778478 10.1016/j.tibs.2015.12.001 PMC4783224

[CR37] Lim SH, Lee HS, Lee CH, Choi CI (2021) Pharmacological activity of Garcinia indica (Kokum): an updated review. Pharmaceuticals 14(12):1338 34959738 10.3390/ph14121338 PMC8708457

[CR38] Liu C, Ho PCL, Wong FC, Sethi G, Wang LZ, Goh BC (2015) Garcinol: current status of its anti-oxidative, anti-inflammatory and anti-cancer effects. Cancer Lett 362(1):8–14 25796441 10.1016/j.canlet.2015.03.019

[CR39] Liu H, Gan F, Jin S, Li J, Chen Y, Yang G (2017) Acylphloroglucinol and tocotrienol derivatives from the fruits of Garcinia multiflora. RSC Adv 7(47):29295–29301 10.1039/C7RA04208F

[CR40] Lu L, Mullins CS, Schafmayer C, Zeißig S, Linnebacher M (2021) A global assessment of recent trends in gastrointestinal cancer and lifestyle-associated risk factors. Cancer Commun 41(11):1137–1151 10.1002/cac2.12220 PMC8626600 34563100

[CR41] Lu Q, Kou D, Lou S, Ashrafizadeh M, Aref AR, Canadas I, Han P (2024) Nanoparticles in tumor microenvironment remodeling and cancer immunotherapy. J Hematol Oncol 17(1):16. 10.1186/s13045-024-01535-8 38566199 10.1186/s13045-024-01535-8 PMC10986145

[CR42] Majeed M, Bani S, Bhat B, Pandey A, Mundkur L, Neupane P (2018) Safety profile of 40% garcinol from Garcinia indica in experimental rodents. Toxicol Rep 5:750–758 29984188 10.1016/j.toxrep.2018.06.009 PMC6031240

[CR43] Mane A, Sawant AA, Sonawane SP, Vidyapeeth BK K (2012) Comparative study of physical properties of kokum rind powder prepared by hammer mill and pulverizer. A Peer-Reviewed Multi-Disciplinary International Journal, p 39

[CR44] Mármol I, Sánchez-de-Diego C, Pradilla Dieste A, Cerrada E, Yoldi R, M. J (2017) Colorectal carcinoma: a general overview and future perspectives in colorectal cancer. Int J Mol Sci 18(1):197 28106826 10.3390/ijms18010197 PMC5297828

[CR45] McGuigan A, Kelly P, Turkington RC, Jones C, Coleman HG, McCain RS (2018) Pancreatic cancer: a review of clinical diagnosis, epidemiology, treatment and outcomes. World J Gastroenterol 24(43):4846 30487695 10.3748/wjg.v24.i43.4846 PMC6250924

[CR46] Noreen S, Khan Naizi M, Tufail T, Hassan F, Awuchi CG (2023) Nutraceutical, functional, and therapeutic properties of Garcinia Cambogia: a review. Int J Food Prop 26(1):729–738 10.1080/10942912.2023.2178458

[CR47] Ohnishi H, Asamoto M, Tujimura K, Hokaiwado N, Takahashi S, Ogawa K, Shirai T (2004) Inhibition of cell proliferation by nobiletin, a dietary phytochemical, associated with apoptosis and characteristic gene expression, but lack of effect on early rat hepatocarcinogenesis in vivo. Cancer Sci 95(12):936–942 15596041 10.1111/j.1349-7006.2004.tb03180.x PMC11158043

[CR48] Pandita A, Kumar B, Manvati S, Vaishnavi S, Singh SK, Bamezai RN (2014) Synergistic combination of gemcitabine and dietary molecule induces apoptosis in pancreatic cancer cells and down regulates PKM2 expression. PLoS ONE, 9(9), e107154 10.1371/journal.pone.0107154 PMC4157832 25197966

[CR49] Parasramka MA, Gupta SV (2011) Garcinol inhibits cell proliferation and promotes apoptosis in pancreatic adenocarcinoma cells. Nutr Cancer 63(3):456–465 21462088 10.1080/01635581.2011.535962

[CR50] Parasramka MA, Gupta SV (2012) Synergistic effect of garcinol and curcumin on antiproliferative and apoptotic activity in pancreatic cancer cells. Journal of oncology, 2012 10.1155/2012/709739 PMC3366245 22685460

[CR51] Paskeh MDA, Entezari M, Mirzaei S, Zabolian A, Saleki H, Naghdi MJ, Ashrafizadeh M (2022) Emerging role of exosomes in cancer progression and tumor microenvironment remodeling. J Hematol Oncol 15(1):83. 10.1186/s13045-022-01305-4 35765040 10.1186/s13045-022-01305-4 PMC9238168

[CR52] Ranjbarnejad T, Saidijam M, Tafakh MS, Pourjafar M, Talebzadeh F, Najafi R (2017) Garcinol exhibits anti-proliferative activities by targeting microsomal prostaglandin E synthase-1 in human colon cancer cells. Hum Exp Toxicol 36(7):692–700 27481098 10.1177/0960327116660865

[CR53] Rao Gajula SN, Talari S, Chilvery S, Godugu C, Sonti R (2023) A unique in vivo pharmacokinetic profile, in vitro metabolic stability and hepatic first-pass metabolism of garcinol, a promising novel anticancer phytoconstituent, by liquid chromatography–mass spectrometry. RPS Pharm Pharmacol Rep 2(2):rqad017 10.1093/rpsppr/rqad017

[CR54] Rawla P, Sunkara T, Gaduputi V (2019a) Epidemiology of pancreatic cancer: global trends, etiology and risk factors. World J Oncol 10(1):10 30834048 10.14740/wjon1166 PMC6396775

[CR55] Rawla P, Sunkara T, Thandra KC, Barsouk A (2019b) Epidemiology of gallbladder cancer. Clin Experimental Hepatol 5(2):93–102. 10.5114/ceh.2019.85166 10.5114/ceh.2019.85166 PMC6728871 31501784

[CR56] Rizvi, S. M. D., Almazni, I. A., Moawadh, M. S., Alharbi, Z. M., Helmi, N., Alqahtani,L. S., … Tiwari, R. K. (2024). Targeting NF-κB signaling cascades of glioblastoma by a natural benzophenone, garcinol, via in vitro and molecular docking approaches. Frontiers in Chemistry, 12, 1352009 10.3389/fchem.2024.1352009 PMC10904546 38435669

[CR57] Rumgay, H., Arnold, M., Ferlay, J., Lesi, O., Cabasag, C. J., Vignat, J., … Soerjomataram,I. (2022). Global burden of primary liver cancer in 2020 and predictions to 2040.Journal of hepatology, 77(6), 1598–1606 10.1016/j.jhep.2022.08.021 PMC9670241 36208844

[CR58] Saadat N (2013) Anti-cancer effects of garcinol in pancreatic cancer transgenic mouse model. Wayne State University

[CR59] Saadat N, Gupta SV (2012) Potential role of garcinol as an anticancer agent. Journal of oncology, 2012 10.1155/2012/647206 PMC3382268 22745638

[CR60] Sawicki T, Ruszkowska M, Danielewicz A, Niedźwiedzka E, Arłukowicz T, Przybyłowicz KE (2021) A review of colorectal cancer in terms of epidemiology, risk factors, development, symptoms and diagnosis. Cancers, 13(9), 2025 10.3390/cancers13092025 PMC8122718 33922197

[CR61] Schobert R, Biersack B (2019) Chemical and biological aspects of garcinol and isogarcinol: recent developments. Chem Biodivers, 16(9), e1900366 10.1002/cbdv.201900366 31386266

[CR62] Sethi G, Chatterjee S, Rajendran P, Li F, Shanmugam MK, Wong KF, Kundu TK (2014) Inhibition of STAT3 dimerization and acetylation by garcinol suppresses the growth of human hepatocellular carcinoma in vitro and in vivo. Mol Cancer 13:1–14 24655440 10.1186/1476-4598-13-66 PMC3998115

[CR63] Sheikh, I., Sharma, V., Tuli, H. S., Aggarwal, D., Sankhyan, A., Vyas, P., … Bishayee, A. (2020). Cancer chemoprevention by flavonoids, dietary polyphenols and terpenoids.Biointerface Res Appl Chem, 11(1), 8502–8537

[CR64] Sitarz R, Skierucha M, Mielko J, Offerhaus GJA, Maciejewski R, Polkowski WP (2018) Gastric cancer: epidemiology, prevention, classification, and treatment. Cancer Manage Res, 239–248 10.2147/CMAR.S149619 PMC5808709 29445300

[CR65] Staebler S, Hoechst S, Thongmao A, Schneider N, Bosserhoff AK, Kuphal S (2024) The role of T-Cadherin (CDH13) in treatment options with Garcinol in Melanoma. Cancers 16(10):1853 38791932 10.3390/cancers16101853 PMC11119778

[CR66] Stark TD, Salger M, Frank O, Balemba OB, Wakamatsu J, Hofmann T (2015) Antioxidative compounds from Garcinia Buchananii stem bark. J Nat Prod 78(2):234–240 25625705 10.1021/np5007873

[CR67] Sung H, Ferlay J, Siegel RL, Laversanne M, Soerjomataram I, Jemal A, Bray F (2021) Global cancer statistics 2020: GLOBOCAN estimates of incidence and mortality worldwide for 36 cancers in 185 countries. Cancer J Clin 71(3):209–249 10.3322/caac.21660 33538338

[CR68] Then EO, Lopez M, Saleem S, Gayam V, Sunkara T, Culliford A, Gaduputi V (2020) Esophageal cancer: an updated surveillance epidemiology and end results database analysis. World J Oncol 11(2):55 32284773 10.14740/wjon1254 PMC7141161

[CR69] Uhlenhopp DJ, Then EO, Sunkara T, Gaduputi V (2020) Epidemiology of esophageal cancer: update in global trends, etiology and risk factors. Clin J Gastroenterol 13(6):1010–1021 32965635 10.1007/s12328-020-01237-x

[CR70] Ullah MF, Ahmad A (eds) (2016) Critical dietary factors in cancer chemoprevention. Springer, Switzerland

[CR71] Unnikrishnan Meenakshi D, Narde GK, Ahuja A, Al Balushi K, Francis AP, Khan SA (2024) Therapeutic applications of Nanoformulated Resveratrol and Quercetin Phytochemicals in Colorectal Cancer—An. Updated Rev Pharm 16(6):761 10.3390/pharmaceutics16060761 PMC11206904 38931884

[CR72] Wang J, Wu M, Zheng D, Zhang H, Lv Y, Zhang L, Xu HX (2020) Garcinol inhibits esophageal cancer metastasis by suppressing the p300 and TGF-β1 signaling pathways. Acta Pharmacol Sin 41(1):82–92 31371781 10.1038/s41401-019-0271-3 PMC7471459

[CR73] Wang MM, Coupland SE, Aittokallio T, Figueiredo CR (2023) Resistance to immune checkpoint therapies by tumour-induced T-cell desertification and exclusion: key mechanisms, prognostication and new therapeutic opportunities. Br J Cancer 129(8):1212–1224 37454231 10.1038/s41416-023-02361-4 PMC10575907

[CR74] Wang S, Zheng R, Li J, Zeng H, Li L, Chen R, He J (2024) Global, regional, and national lifetime risks of developing and dying from gastrointestinal cancers in 185 countries: a population-based systematic analysis of GLOBOCAN. Lancet Gastroenterol Hepatol 9(3):229–237 38185129 10.1016/S2468-1253(23)00366-7 PMC10849975

[CR75] Zhao Z, Liu W (2020) Pancreatic cancer: a review of risk factors, diagnosis, and treatment. Technol Cancer Res Treat 19:1533033820962117 33357065 10.1177/1533033820962117 PMC7768873

[CR76] Zheng D, Zhang H, Zheng CW, Lao YZ, Xu DQ, Xiao LB, Xu HX (2017) Garciyunnanimines A–C, novel cytotoxic polycyclic polyprenylated acylphloroglucinol imines from Garcinia Yunnanensis. Org Chem Front 4(11):2102–2108 10.1039/C7QO00485K

[CR77] Zheng Y, Guo C, Zhang X, Wang X, Ma AΗ (2020) Garcinol acts as an antineoplastic agent in human gastric cancer by inhibiting the PI3K/AKT signaling pathway. Oncol Lett 20(1):667–676 32565991 10.3892/ol.2020.11585 PMC7285879

[CR78] Zhu SL, Qi M, Chen MT, Lin JP, Huang HF, Deng LJ, Zhou XW (2024) A novel DDIT3 activator dehydroevodiamine effectively inhibits tumor growth and tumor cell stemness in pancreatic cancer. Phytomedicine 128:155377 38503154 10.1016/j.phymed.2024.155377

